# *Fagonia cretica* L. and Redox Homeostasis: An Integrative Review of Phytochemistry, Redox-Sensitive Signaling, and Pharmacological Potential

**DOI:** 10.3390/ph19071036

**Published:** 2026-07-03

**Authors:** Asad Abbas, Saeed Vohra, Ralf Weiskirchen, Hameeza Mushtaq, Adnan Amjad, Arooma Tabassum, Shehnshah Zafar, Anis Ahmad Chaudhary, Abdulrahman Mohammed Alhudhaibi, Bipindra Pandey

**Affiliations:** 1 Department of Human Nutrition, Faculty of Food Science and Nutrition, Bahauddin Zakariya University, Multan 60800, Pakistan; asadabbaskhichi@gmail.com (A.A.); hameezamushtaq@gmail.com (H.M.); adnanamjad@bzu.edu.pk (A.A.); shah8942123@gmail.com (S.Z.); 2 Department of Anatomy and Physiology, College of Medicine, Imam Mohammad Ibn Saud Islamic University, Riyadh 11623, Saudi Arabia; smvsaeed@imamu.edu.sa; 3 Institute of Molecular Pathobiochemistry, Experimental Gene Therapy and Clinical Chemistry (IFMPEGKC), RWTH University Hospital Aachen, D-52074 Aachen, Germany; 4 Department of Chemistry, Government College University, Lahore 54000, Pakistan; arooma.tabassum@outlook.com; 5 Department of Biology, College of Science, Imam Mohammad Ibn Saud Islamic University (IMSIU), Riyadh 11623, Saudi Arabia; aachaudhary@imamu.edu.sa (A.A.C.); amalhudhaibi@imamu.edu.sa (A.M.A.); 6 Department of Pharmacy, Madan Bhandari Academy of Health Sciences, Hetauda 44107, Nepal

**Keywords:** bioactive compounds, *Fagonia cretica* L., pharmacological activity, phytochemistry, reactive oxygen species, redox homeostasis, oxidative stress

## Abstract

Redox homeostasis is the balance between oxidative processes and antioxidant defenses and is fundamental to cellular integrity. This review critically synthesizes current evidence on the phytochemical composition, redox-modulating mechanisms, and therapeutic bioactivities of *Fagonia cretica* L. (*F. cretica*), with the aim of evaluating its translational potential as a natural antioxidant and anticancer agent. *F. cretica* has emerged as a phytochemically rich candidate containing highly bioactive secondary metabolite for redox-targeted therapeutic applications. Its diverse secondary metabolite profile, including alkaloids, flavonoids, tannins, saponins, terpenoids, glycosides, and phenolic compounds, confers broad biological activity. Bioactive constituents, particularly kaempferol, catechin, quercetin, and arbutin, directly neutralize reactive oxygen species (ROS) and modulate inflammatory pathways through inhibition of COX-1, COX-2, and nitric oxide production. These compounds influence important major ROS-sensitive redox signaling pathways: activation of the Keap1/Nrf2/ARE axis to upregulate cytoprotective genes such as HO-1, NQO1, and GCL, suppression of the NF-κB pathway to attenuate pro-inflammatory cytokine transcription, including TNF-α, IL-1β, and IL-6, and interference with the MAPK-PI3K/Akt cascade to disrupt aberrant cancer cell survival and proliferation. Bioactive compound-rich extracts of *F. cretica* exhibit anticancer activity in MCF-7 breast cancer cells by inducing DNA damage, cell cycle arrest, and apoptotic signaling through the FOXO3a/p53 pathways. Similar effects have been reported in colorectal (HCT-116) and prostate (PC-3) cancer cells through DNA (cytosine-5)-methyltransferase 1 (DNMT1) downregulation, oxidative stress induction, and ER-β activation. Moreover, these extracts demonstrate cytotoxic effects in HepG2 and Caco-2 intestinal cancer cells, often associated with topoisomerase inhibition and caspase activation. Despite encouraging preclinical evidence, systematic studies encompassing pharmacokinetic profiling, toxicological characterization, and human clinical trials remain essential to translate these findings into safe, evidence-based therapeutic applications.

## 1. Introduction

Redox homeostasis is a biological process that maintains the balance between the production of reactive oxygen species (ROS) and antioxidant defenses in cells [1]. ROS are continually formed as byproducts of normal aerobic metabolism, such as superoxide (O_2_^−^), hydrogen peroxide (H_2_O_2_), and hydroxyl radicals (OH•) [2,3]. At the physiological level, these molecules act as messengers in intracellular signaling pathways involved in cell proliferation, cell death, immune system regulation, and metabolic homeostasis [4]. Cells detect and respond to changes in intracellular ROS levels through several redox-sensitive signaling pathways. Among the most extensively studied are the Keap1/Nrf2/ARE pathway, which regulates cytoprotective antioxidant genes, the NF-κB signaling pathway, which controls inflammatory gene expression, and signaling cascades involving MAPK and PI3K/Akt that influence cell survival and proliferation. Oxidation of Keap1 cysteine residues results in the release of Nrf2, leading to the transcription of more than 200 cytoprotective genes, including GCL, HO-1, and NQO1 [5]. Elevated ROS levels can promote activation of the NF-κB signaling pathway through redox-dependent modulation of upstream kinases such as IκB kinase (IKK). Activation of this pathway leads to nuclear translocation of NF-κB and subsequent transcription of pro-inflammatory cytokines, including TNF-α, IL-1β, and IL-6 [6,7]. ROS can also influence cell survival pathways by modulating redox-sensitive signaling proteins. For example, ROS may transiently oxidize the catalytic cysteine residue of the tumor suppressor phosphatase PTEN, resulting in reversible inhibition of its phosphatase activity. This redox-dependent modification can enhance PI3K/Akt signaling and promote cellular survival and proliferation under certain physiological or pathological conditions. However, this regulatory mechanism is highly context-dependent and reversible, reflecting the dynamic nature of intracellular redox signaling [8]. This delicate equilibrium becomes disturbed when an imbalance occurs, either through chronic overproduction of ROS leading to oxidative stress or through excessive antioxidant activity leading to reductive stress. Consequently, cells are progressively damaged, forming the basis of the pathogenesis of major diseases including cancer, cardiovascular diseases, type 2 diabetes, and liver diseases [7].

Medicinal plants have long been recognized as important sources of bioactive compounds with the potential to influence redox biology, a property that extends beyond simple free-radical scavenging. *F. cretica* is a spiny, xerophytic shrub belonging to the family *Zygophyllaceae*, occuring throughout the Mediterranean Basin, North Africa, the Arabian Peninsula, and parts of the Indian subcontinent, typically in arid and semi-arid environments [9]. It is well established in traditional medicine systems such as Ayurveda, Unani, and regional ethnobotanical practices, having been used for centuries to treat inflammatory diseases, liver disorders, skin diseases, fever, and cancer [10]. These traditional uses provide a strong historical basis for current pharmacological research. Phytochemical analyses have shown that *F. cretica* is rich in structurally complex secondary metabolites. These include flavonoids such as quercetin, kaempferol, luteolin, apigenin, and rutin, triterpenoid saponins and glycosides, including oleanane- and ursane-type glycosides such as hederagenin-, oleanolic acid-, and 27-hydroxyoleanolic acid-based glycosides. Moreover, alkaloids such as harmine and harmaline, iridoid glycosides, phenolic acids such as gallic acid, caffeic acid, and chlorogenic acid, are included, as well as sterols and tannins [11]. Importantly, many of these constituents possess the ability to interact with redox-sensitive molecular targets, suggesting that *F. cretica* may represent a promising botanical candidate for further investigation within the emerging paradigm of precision redox medicine.

The pharmacological properties attributed to the bioactive compounds of *Fagonia* species are increasingly being elucidated through studies of specific molecular mechanisms related to redox regulation. Suggested replacement: At the broader phytochemical level, flavonoids and saponins from medicinal plants have been reported to influence apoptosis-related redox signaling in cancer models, including mitochondrial membrane depolarization, cytochrome c release, caspase activation, and suppression of NF-κB-mediated anti-apoptotic signaling. These findings provide mechanistic background for interpreting redox-active phytochemicals; however, they should not be interpreted as compound-specific evidence for *F. cretica* unless the cited studies directly examined *F. cretica* water extracts, fractions, or isolated constituents [12,13]. These effects are consistent with selective pro-oxidant cytotoxicity that exploits the increased ROS vulnerability of malignant cells. The hepatoprotective activity of *F. cretica* has been associated with inhibition of CYP2E1-mediated oxidative metabolism, reduction in lipid peroxidation products such as 4-HNE and MDA, restoration of hepatic glutathione reserves, and inhibition of TGF-β-induced activation of hepatic stellate cells. Together, these actions interrupt the redox-inflammatory pathway that drives the progression from steatosis to fibrosis and cirrhosis [14]. Anti-inflammatory effects include suppression of the NF-κB pathway and a subsequent decrease in the secretion of TNF-α, IL-1β, and IL-6, as well as inhibition of COX-2 and modulation of the NLRP3 inflammation, thereby disrupting the ROS–inflammation feed-forward cycle responsible for maintaining chronic tissue injury [15]. The antioxidant activity of *F. cretica* compounds appears to be mediated partly by direct radical scavenging, attributed to the phenolic hydroxyl groups [16,17], but is primarily mediated indirectly through activation of the Nrf2 pathway. This activation leads to upregulation of endogenous antioxidant enzymes such as heme oxygenase-1 (HO-1), thioredoxin reductase, NQO1, and the glutathione synthesis enzymes GCL and GSS [18,19]. This hormetic mechanism enhances antioxidant capacity in cells far more effectively than simple radical scavenging and is now recognized as a principal mechanism of action for many plant polyphenols.

Therefore, a review that addresses these gaps and critically integrates current knowledge on *F. cretica* within the modern framework of redox homeostasis is warranted. The present review provides a comprehensive overview of the phytochemical profile of *F. cretica*, the molecular mechanisms by which its principal bioactive compounds influence redox-sensitive pathways in cancer, liver disorders, inflammatory diseases, and antioxidant systems, and a critical evaluation of the quality and translational relevance of existing experimental evidence. This review also highlights key research gaps that must be addressed to advance the field. These include conducting mechanistic studies at the level of individual compounds, employing validated redox biomarker panels, developing pharmacological models tailored to specific disease stages, performing comprehensive toxicological assessments, and designing well-structured in vivo studies. The aim of this review is to provide a scientifically grounded framework for the future development of *F. cretica* pharmacology and to clarify its true therapeutic potential, as well as the challenges that must be addressed before the medicinal potential of this plant can be responsibly translated into clinical applications.

## 2. Literature Search Strategy and Evidence Sources

The literature included in this narrative review was identified through searches of major scientific databases, including PubMed/MEDLINE, Scopus, Web of Science, ScienceDirect, and Google Scholar. The databases were searched from their inception up to January 2026. Search terms included ‘*Fagonia cretica*’, ‘*Fagonia*’, ‘phytochemistry’, ‘saponins’, ‘flavonoids’, ‘phenolics’, ‘triterpenoids’, ‘redox homeostasis’, ‘oxidative stress’, ‘antioxidant’, ‘anti-inflammatory’, ‘anticancer’, ‘antidiabetic’, ‘hepatoprotective’, and ‘antimicrobial’, combined using Boolean operators where appropriate. The thematic scope included studies reporting nutritional composition, phytochemical characterization, redox-sensitive signaling, and pharmacological activities of *F. cretica*. Studies were included if they reported original experimental findings or directly relevant review evidence on *F. cretica* or clearly identified constituents of the species. Studies were excluded if they did not concern *F. cretica*, lacked extract- or compound-level information, were duplicate records, were conference abstracts without accessible data, or involved multi-plant preparations without separable *F. cretica*-specific findings. General mechanistic studies on flavonoids, saponins, or redox signaling were used only as contextual evidence and were not treated as species-specific evidence for *F. cretica*.

## 3. Redox Homeostasis as a Central Regulator of Human Health

**Figure 1:**
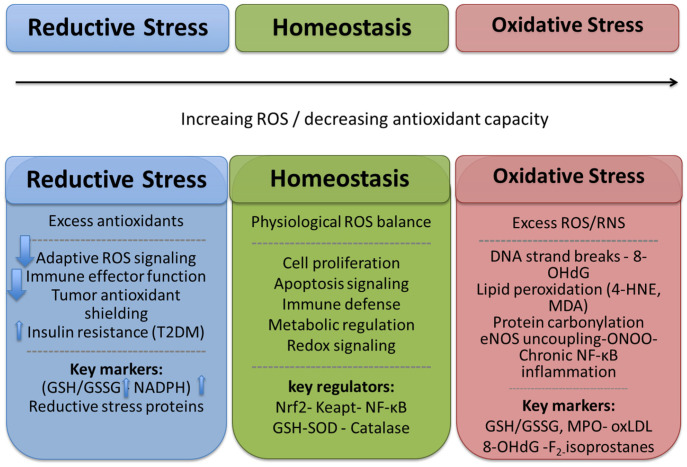
Redox homeostasis as a central regulator of human health.

Redox homeostasis is defined as the dynamic balance between ROS, such as superoxide (O_2_^−^), hydrogen peroxide (H_2_O_2_), and hydroxyl radicals (OH•), and cellular antioxidant defenses, including thioredoxin (Trx), superoxide dismutase (SOD), and catalase (CAT) [20]. Physiological levels of ROS are essential for intracellular signaling processes that regulate cell proliferation, apoptosis, immune activation, and metabolic homeostasis (Figure 1).

There are three commonly studied pathways that detect redox perturbations: (1) ROS oxidize Keap1, leading to the release of Nrf2, which activates the transcription of cytoprotective genes, including GCL, HO-1, and NQO1; (2) ROS activate IκB kinase, thereby releasing NF-κB to drive the expression of TNF-α, IL-1β, and IL-6, creating a self-sustaining inflammatory loop; and (3) ROS can also modulate signaling pathways by reversibly oxidizing redox-sensitive proteins such as PTEN and other protein tyrosine phosphatases. Oxidation of critical cysteine residues can transiently inhibit their phosphatase activity, thereby enhancing downstream signaling through pathways such as PI3K/Akt that regulate cell survival and proliferation [21,22].

Moreover, cellular redox balance exists along a continuum ranging from reductive stress to oxidative stress, with redox homeostasis representing the optimal physiological equilibrium between ROS production and antioxidant defense systems. Reductive stress results from excessive antioxidant activity and diminished ROS signaling, leading to impaired immune function, insulin resistance, and altered cellular metabolism. In contrast, oxidative stress occurs when the generation of ROS and reactive nitrogen species (RNS) exceeds the antioxidant capacity, causing DNA damage, lipid peroxidation, protein carbonylation, endothelial nitric oxide synthase (eNOS) uncoupling, and chronic NF-κB-mediated inflammation [23]. Under normal conditions, redox homeostasis supports cell proliferation, apoptosis signaling, immune defense, metabolic regulation, and intracellular signaling pathways [24,25]. Key biomarkers associated with these states include the GSH/GSSG ratio, NADPH, Nrf2, 8-hydroxy-2′-deoxyguanosine (8-OHdG), F2-isoprostanes, myeloperoxidase (MPO), malondialdehyde (MDA), and oxidized low-density lipoprotein (LDL).

## 4. Nutritional Composition of *F. cretica*

The nutritional profile of *F. cretica* exhibits a balanced distribution of macronutrients, structural carbohydrates, and essential micronutrients, indicating its potential role in supporting fundamental physiological processes and maintaining metabolic homeostasis. It contains moisture (10.41%), crude fiber (14.57%), protein (9.34%), and lipids (4.63%). It also contains carbohydrates, including glucose, sucrose, soluble sugars, and polysaccharides, with a total carbohydrate content of 622.4 mg/g dry weight. Its energy value is 327.99 kcal/100 g, suggesting a moderate caloric content. The carbohydrate content suggests its potential utility in providing both rapid and sustained energy, thereby supporting ATP generation and metabolic demands. Its energy value of 327.99 kcal/100 g further reinforces its contribution to dietary energy intake [26].

The mineral composition of *F. cretica* also contributes to its nutritional significance. Essential elements such as potassium (12.09 mg/g), sodium (13.09 mg/g), calcium (10.69 mg/g), and magnesium (7.19 mg/g) occur in substantial quantities and play important roles in osmotic balance, neuromuscular function, and enzyme activation. Trace elements such as iron (1.13 mg/g), zinc (0.07 mg/g), copper (0.31 mg/g), and manganese (0.09 mg/g) serve as essential cofactors for numerous enzymatic systems [26]. These minerals are important components of endogenous antioxidant defense mechanisms, including the activation of key enzymes such as superoxide dismutase (SOD), catalase, and glutathione-related pathways, thereby indirectly contributing to the regulation of ROS. Owing to its rich nutrient composition, *F. cretica* may be considered a promising functional food ingredient.

*F. cretica* contains a wide array of phytoconstituents, including active antioxidant compounds. The total phenolic content (TPC) and total flavonoid content (TFC) were reported as 2.4 mg GAE/g and 0.18 mg GAE/g, respectively, while the antioxidant activity was estimated to be 1.40 mg AEAC/g. The presence of these bioactive compounds suggests potential antioxidant activity capable of counteracting oxidative stress [27].

*F. cretica* is a nutritious, edible, and medicinal herb containing alkaloids (3.32–4.43 mg/g), tannins (6.66–12.44 mg/g), total phenolics (4.38–9.62 mg/g), saponins (5.87–8.14 mg/g), and flavonoids (5.11–7.16 mg/g). The plant also exhibits significant antioxidant activity of approximately 1.40 mg/g [26]. It is a rich source of various bioactive molecules of medicinal importance. The active constituents of *F. cretica* include alkaloids, fatty acids, triterpenoids, and steroids. The major bioactive constituents include quinovic acid and its derivatives, such as quinovic acid-3β-O-β-D-glycopyranoside and quinovic acid-3β-O-β-D-glucopyranosyl-(28→1)-β-D-glucopyranosyl ester. Sterols and related compounds reported in the plant include stigmasterol, linoleic acid, octacosanoic acid, methyl triadecanoate, β-amyrin acetate, taraxerol, oleanolic aldehyde acetate, and triacontanoic acid. Other important bioactive molecules include diosgenin, cryptogenin, lanosterol, harmine, betulin, and campesterol. These compounds are associated with antioxidant, anti-inflammatory, and antimicrobial activities [28,29,30].

The aqueous extract of *F. cretica* contains phenolic acids and flavonoids. HPLC analysis by Kamran et al. revealed the presence of quercetin (7.82 ppm), gallic acid (2.43 ppm), vanillic acid (5.64 ppm), benzoic acid (8.66 ppm), *m*-coumaric acid (0.97 ppm), and sinapic acid (1.64 ppm). These compounds possess antioxidant, anti-inflammatory, and antimicrobial activities, which may contribute to the therapeutic effects of the herb [31].

**Figure 2:**
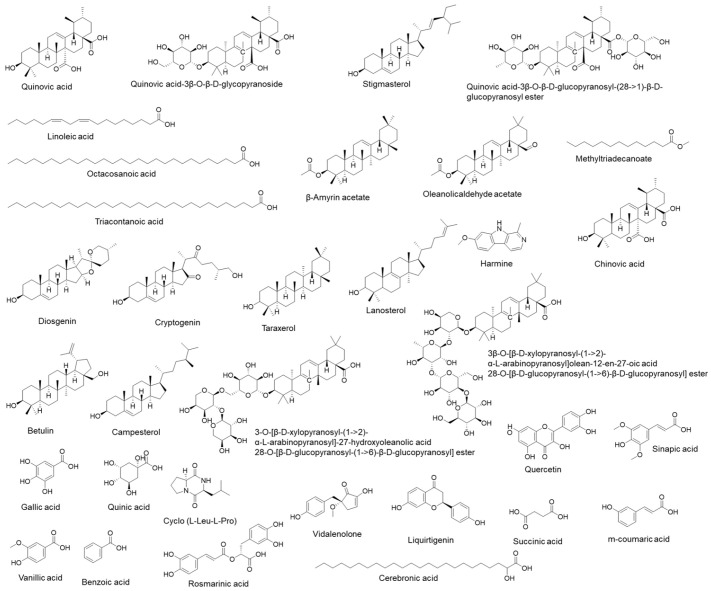
Major bioactive phytoconstituents present in the *F. cretica* [28,29,30,31,32].

Similarly, the methanolic extract of *F. cretica* is rich in phenolic acids and flavonoids. HPLC analysis detected quercetin (8.28 ppm), gallic acid (2.52 ppm), vanillic acid (6.72 ppm), benzoic acid (11.21 ppm), *m*-coumaric acid (2.14 ppm), cinnamic acid (4.25 ppm), and sinapic acid (2.01 ppm) [31]. These compounds have been reported to possess potent antioxidant, anti-inflammatory, and antibacterial effects, demonstrating the pharmaceutical potential of the extract (Table 1 and Figure 2). Variations in extraction solvent, plant part, geographical origin, and phytochemical composition may substantially affect the redox-modulating efficacy of *F. cretica*. Aqueous, methanolic, ethanolic, and other solvent-based extracts, as well as nanoparticle-based preparations, differ in their phenolic, flavonoid, saponin, alkaloid, and triterpenoid contents, which may influence antioxidant capacity, enzyme inhibition, inflammatory signaling, and cytotoxic effects. Therefore, future studies should clearly report botanical authentication, plant part used, extraction solvent, extraction yield, marker compounds, dosage, assay conditions, and redox-specific biomarkers to improve reproducibility and facilitate mechanistic interpretation.

## 5. Health Promoting Mechanisms via Redox Homeostasis

### 5.1. Antioxidant Mechanism

NADPH oxidases, specifically nicotinamide adenine dinucleotide phosphate oxidase 2 (NOX2) and nicotinamide adenine dinucleotide phosphate oxidase 4 (NOX4), are among the most important vascular ROS-producing enzymes in cardiovascular disease and are stimulated by angiotensin II, oscillatory shear stress, LDL, and hyperglycemia [33]. The driving pathological event is kinetic because superoxide is a highly reactive species that reacts three times more rapidly with endothelial nitric oxide (NO) than with SOD to produce peroxynitrite (ONOO^−^). Peroxynitrite oxidizes the eNOS cofactor tetrahydrobiopterin (BH_4_) and promotes eNOS uncoupling, converting eNOS into a net superoxide producer and creating a self-amplifying vascular oxidative cycle [34]. The consequences include endothelial dysfunction (impaired vasorelaxation and increased vascular cell adhesion molecule-1 (VCAM-1), intercellular adhesion molecule-1 (ICAM-1), and E-selectin expression), macrophage uptake of oxidized LDL via scavenger receptors leading to foam cell formation and atherogenesis, and cardiac remodeling characterized by increased activation of matrix metalloproteinases (MMPs), cardiomyocyte apoptosis, and mitochondrial ROS-mediated pathological hypertrophy [35,36]. The succinate–Complex II–reverse electron transport mechanism produces a surge of mitochondrial ROS during ischemia–reperfusion, which can paradoxically exacerbate injury upon reperfusion of the coronary arteries [37]. MPO is both a mechanistic driver and an independent clinical predictor of cardiovascular events. In contrast, dietary polyphenols reduce cardiovascular risk via Nrf2-mediated hormesis rather than direct radical scavenging, which may explain their limited efficacy in cardiovascular trials where antioxidant vitamins are administered individually in high doses [38].

Phytochemicals of *F. cretica* include flavonoids (quercetin, kaempferol, luteolin, apigenin), alkaloids (harmine, vasicine, sphaerophysine), triterpenoid saponins (oleanolic acid and ursolic acid derivatives), phenolic acids (gallic acid, ferulic acid, protocatechuic acid), and diverse terpenoids [28,29,30]. These compounds can interact with ROS, including superoxide anions and hydroxyl radicals, and may contribute to the reduction in oxidative damage. However, in biological systems their antioxidant effects are believed to arise primarily from modulation of endogenous defense mechanisms rather than direct scavenging of all ROS species [39]. At the mechanistic level, the antioxidant properties of *F. cretica* are closely linked to activation of the body’s endogenous cellular defense systems, particularly the nuclear factor erythroid 2–related factor 2 (Nrf2) signaling pathway [40]. Under oxidative or electrophilic stress conditions, certain phytochemicals can modify cysteine residues within the Keap1 protein or interfere with the Keap1-Nrf2 interaction. This process stabilizes Nrf2 and allows its translocation to the nucleus, where it activates antioxidant response element (ARE)-dependent gene expression. In the nucleus, Nrf2 binds to antioxidant response elements (ARE) in DNA, promoting the expression of phase II detoxifying and antioxidant enzymes such as superoxide dismutase (SOD), catalase (CAT), glutathione peroxidase (GPx), and heme oxygenase-1 (HO-1). This coordinated enzymatic response enhances intracellular antioxidant capacity, reduces lipid peroxidation, and restores redox balance [41]. Additionally, *F. cretica* suppresses ROS-mediated activation of pro-inflammatory and pro-apoptotic pathways, including NF-κB signaling, thereby indirectly reducing oxidative stress-induced inflammation and cellular injury. Through this dual mechanism—direct radical scavenging and indirect activation of the Nrf2–ARE antioxidant defense system—the plant protects cells from oxidative stress–induced damage, which is a key mechanism underlying chronic diseases such as cancer, diabetes, and neurodegenerative disorders [42]. At the cellular and molecular levels, *F. cretica* phytochemicals appear to modulate redox homeostasis through complementary antioxidant and context-dependent pro-oxidant mechanisms. Phenolic acids and flavonoids may directly reduce ROS through electron- or hydrogen-donating activity, whereas electrophilic phytochemicals may influence Keap1 cysteine residues, stabilize Nrf2, and promote ARE-dependent transcription of antioxidant enzymes. This response enhances the activity of SOD, CAT, GPx, HO-1, NQO1, and glutathione-related defense systems, thereby reducing lipid peroxidation and oxidative macromolecular damage. In malignant cells, flavonoid- and saponin-rich fractions may increase ROS levels beyond the apoptotic threshold, causing mitochondrial dysfunction, cytochrome c release, and caspase-dependent apoptosis [40,41].

Recent studies have demonstrated that *F. cretica* possesses a wide range of pharmacological properties owing to its rich phytochemical composition, including flavonoids, phenolics, tannins, and saponins. Nanotechnology-based approaches have further enhanced its therapeutic outcomes. For example, PEGylated nanocomposites containing *F. cretica* aqueous extract have shown substantial reductions in oxidative stress, ROS levels, and DNA damage in CCl_4_-induced hepatotoxic rats, significantly improving hepatoprotective effects compared with conventional treatments [43]. The high polysaccharide and dietary fiber contents also suggest a potential role in maintaining redox homeostasis through enhancement of endogenous antioxidant defenses, reduction in ROS accumulation, suppression of oxidative stress-induced inflammatory signaling, and preservation of mitochondrial function. These effects may collectively protect cellular macromolecules and maintain physiological redox balance. Similarly, green-synthesized nanoparticles, including silver, selenium, and zinc oxide nanoparticles produced using *F. cretica* extract, have exhibited strong antioxidant, antimicrobial, and antidiabetic activities. These effects are attributed to enzyme inhibition (α-amylase and α-glucosidase) and improved metabolic profiles in diabetic models [44]. In combination with *Withania coagulans*, *F. cretica* has demonstrated synergistic effects in lowering hyperuricemia by inhibiting xanthine oxidase, promoting uric acid excretion, modulating gut microbiota, and suppressing inflammatory pathways such as TLR4/MyD88/NF-κB and IL-17 in metabolic disorders [45]. Anticancer studies have also shown that *F. cretica* ethanolic extracts possess potent cytotoxic and antiproliferative activities in cancer cell lines such as HepG2 and MCF-7 by inducing apoptosis, inhibiting topoisomerase enzyme activity, and modulating signaling pathways including TNF-α and TGF-β [17]. Additionally, its extracts exhibit anti-inflammatory activity through inhibition of COX-1/COX-2 and suppression of nitric oxide production. Advances in extraction techniques, such as the use of deep eutectic solvents, have further improved the yield and antioxidant potential of its bioactive flavonoids, making the process more efficient and sustainable [46]. Furthermore, *F. cretica* has been shown to modulate drug-metabolizing enzymes (CYP3A4 and UGT2B7), suggesting a potential role in influencing drug metabolism in diabetic patients [31].

### 5.2. Anti-Inflammatory Mechanism

*F. cretica* has demonstrated notable anti-inflammatory potential due to its rich phytochemical profile, including flavonoids (quercetin-like compounds), phenolics, saponins, and alkaloids. Its anti-inflammatory activity is mainly mediated through inhibition of key pro-inflammatory signaling pathways, particularly the NF-κB and MAPK (mitogen-activated protein kinase) pathways. Under inflammatory stimulation, such as lipopolysaccharide (LPS) exposure or oxidative stress, NF-κB normally translocates from the cytoplasm to the nucleus, promoting the transcription of inflammatory mediators such as TNF-α, IL-1β, IL-6, COX-2, and iNOS [47]. Bioactive-rich fractions of *F. cretica* ethanolic extracts suppress the phosphorylation and activation of IKK, thereby preventing the degradation of IκBα and blocking NF-κB nuclear translocation. This results in reduced expression of pro-inflammatory cytokines and enzymes responsible for prostaglandin and nitric oxide production. In parallel, its phytoconstituents modulate the MAPK cascade (ERK, JNK, and p38 pathways), further downregulating inflammatory gene expression and attenuating macrophage activation [31]. Additionally, the plant exhibits strong antioxidant activity by enhancing endogenous antioxidant enzymes such as SOD, CAT, and GPx while reducing ROS generation. This indirectly suppresses inflammation, since oxidative stress is a major trigger of inflammatory signaling. Some studies also suggest modulation of the NLRP3 inflammasome pathway, leading to reduced activation of caspase-1 and decreased maturation of IL-1β [48]. Overall, *F. cretica* exerts a multitargeted anti-inflammatory effect through suppression of NF-κB/MAPK signaling, inhibition of cytokine release, downregulation of COX-2/iNOS expression, and reduction in oxidative stress, making it a promising natural therapeutic candidate for inflammatory disorders. The redox-sensitive signaling pathways reported or mechanistically implicated for *F. cretica* include the Keap1/Nrf2/ARE antioxidant pathway, NF-κB-mediated inflammatory signaling, MAPK cascades involving ERK, JNK, and p38, PI3K/Akt/mTOR-related survival and metabolic signaling, TLR4/MyD88/NF-κB inflammatory signaling, NLRP3 inflammasome activation, and apoptosis-associated p53/FOXO3a/caspase pathways. These pathways provide a mechanistic framework linking the phytochemical profile of *F. cretica* with antioxidant, anti-inflammatory, anticancer, hepatoprotective, and antidiabetic effects [47,48].

**Figure 3:**
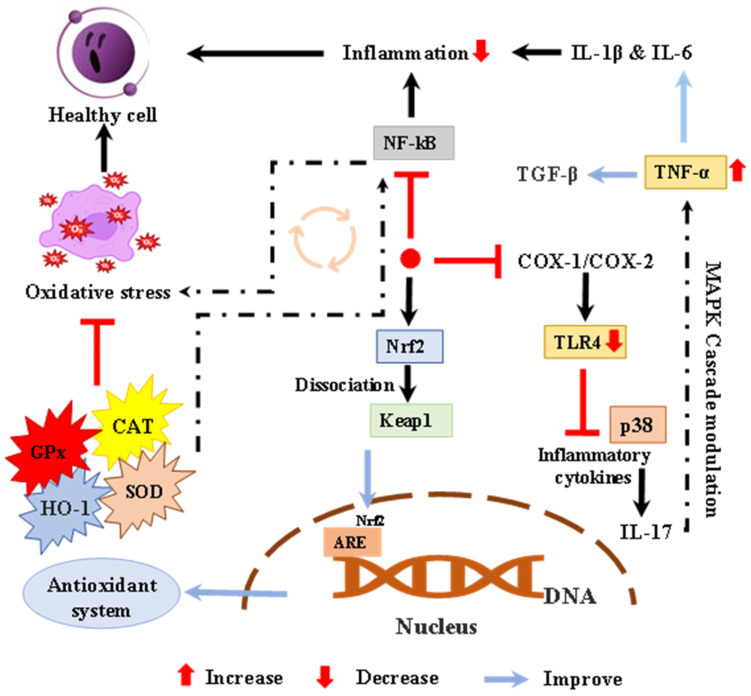
Anti-inflammatory and antioxidant mechanisms of *F. cretica* bioactive compounds involving coordinated Nrf2–NF-κB–TLR4 signaling in redox homeostasis and inflammation resolution. This figure illustrates how redox-active bioactive compounds restore cellular oxidative balance through three coordinated mechanisms. These compounds suppress nuclear factor kappa-B (NF-κB) activation, thereby blocking the inflammatory feed-forward loop and reducing pro-inflammatory cytokines, including tumor necrosis factor-alpha (TNF-α) and transforming growth factor-beta (TGF-β). Simultaneously, downregulation of toll-like receptor 4 (TLR4) reduces p38 mitogen-activated protein kinase (p38 MAPK) phosphorylation, thereby attenuating innate immune-driven inflammation. Most critically, these compounds activate the Nrf2–Keap1 antioxidant pathway, enabling nuclear factor erythroid 2-related factor 2 (Nrf2) to translocate to the nucleus and bind to the antioxidant response element (ARE). This interaction upregulates key cytoprotective enzymes, including heme oxygenase-1 (HO-1), superoxide dismutase (SOD), catalase (CAT), and glutathione peroxidase (GPx). Together, these enzymes neutralize reactive oxygen species, restore the reduced glutathione/oxidized glutathione (GSH/GSSG) redox balance, and decrease oxidative damage markers such as malondialdehyde (MDA) and 8-hydroxy-2′-deoxyguanosine (8-OHdG), thereby restoring redox homeostasis. Directional arrows indicate elevated pathological parameters (↑), therapeutically reduced markers (↓), and restored antioxidant responses (→).

Recent literature highlights the diverse pharmacological potential of *F. cretica*, particularly in metabolic, inflammatory, and cytotoxic diseases. The presence of quinovic acid glycosides, triterpenoids (β-amyrin acetate, taraxerol, betulin, and oleanolic derivatives), phytosterols (stigmasterol, campesterol, diosgenin, and lanosterol), and bioactive fatty acids suggests a significant capacity to maintain redox homeostasis [28,29]. These compounds may act synergistically through ROS scavenging, activation of endogenous antioxidant defense pathways, suppression of oxidative stress-induced inflammatory signaling, and preservation of mitochondrial integrity [49]. In another study, *F. cretica* different solvent extract exhibited potent antioxidant, anti-inflammatory, and cytotoxic activities, including significant inhibition of COX-1 and COX-2 enzymes, induction of apoptosis through caspase-9 activation, and strong cytotoxic effects against cancer cell lines (HepG2, MCF-7, and Caco-2). Metabolic and in silico analyses further confirmed the presence of bioactive compounds with high binding affinity to inflammation- and cancer-related targets [50]. Additionally, earlier research on indigenous medicinal plants reported that *F. cretica* possesses notable anticoagulant activity, along with anti-inflammatory and analgesic properties, supporting its traditional medicinal use [27,51]. The integrated antioxidant and anti-inflammatory signaling network proposed for *F. cretica* is illustrated in Figure 3. The presence of phenolic compounds (2.4 mg GAE/g) and flavonoids (0.18 mg GAE/g), together with measurable antioxidant activity (1.40 mg AEAC/g), suggests a potential role in maintaining redox homeostasis [26]. These bioactive constituents may exert free radical-scavenging effects, enhance endogenous antioxidant defense systems, attenuate oxidative stress-induced inflammatory signaling, and protect cellular macromolecules from oxidative damage, thereby contributing to overall cellular and metabolic homeostasis.

### 5.3. Anti-Cancer Mechanism

The relationship between redox biology and cancer is complex and highly stage-dependent. During tumor initiation, excessive ROS can induce oxidative DNA damage, genomic instability, and oncogenic mutations. However, during tumor progression, many cancer cells adapt to oxidative stress by activating antioxidant pathways such as Nrf2, which enables them to maintain ROS levels that promote proliferation while avoiding apoptotic cell death [20,52]. During later stages of progression, tumor cells take advantage of the constitutive activation of Nrf2—often due to mutations in either Nrf2 or Keap1—to increase the production of glutathione. This maintains moderately elevated ROS levels that support proliferative signaling via the MAPK/PI3K–Akt pathway without triggering apoptotic cell death signaling [53]. This adaptive antioxidant reprogramming contributes directly to resistance to chemotherapy and radiotherapy. Therefore, pro-oxidant interventions, such as radiation, anthracyclines, platinum-based agents, photodynamic therapy, and induction of ferroptosis, must be integrated into rational therapeutic strategies that override tumor antioxidant defenses while simultaneously targeting Nrf2 activity and glutathione synthesis [54].

The anticancer activities of *F. cretica* have been evaluated in various cancer models, and its bioactive compounds exhibit cytotoxic and antiproliferative mechanisms that involve modulation of cellular redox status. It should be noted that the majority of the available data are derived from in vitro cell line studies and computational analyses. Therefore, these findings should be regarded as preliminary and mechanistically hypothesis-generating rather than clinically definitive. Phytochemical analysis of ethanolic extracts of *F. cretica* has revealed a variety of bioactive compounds with antiproliferative potential against HepG2 liver cancer cells, as demonstrated by Arshad et al. using a combined in vitro and in silico approach. HepG2 cells were selected as an appropriate model because hepatocellular carcinoma (HCC) is associated with increased basal oxidative stress levels and dysregulated antioxidant signaling. Therefore, compounds that further disrupt cellular redox balance may sensitize these cells to apoptosis [17]. Extracts of *F. cretica* exert inhibitory effects on the proliferation of colorectal and prostate cancer cells by inducing oxidative stress, downregulating the expression of DNA methyltransferase 1 (DNMT1), and upregulating the expression of estrogen receptor beta (ER-β) [55]. The induction of oxidative stress as a mechanism is particularly significant because it directly links redox-active phytochemicals to tumor cell death. The downregulation of DNMT1 also suggests that the extract may influence epigenetic regulation in cancer cells, either as a direct effect of the extract or as a consequence of oxidative stress. Ahmed et al. reported that silver nanoparticles synthesized from methanolic extracts of *F. cretica* can induce apoptosis in cancer cells in a dose-dependent manner. Both the crude extract and the nanoparticle formulation were cytotoxic, although the nanoparticle formulation showed greater potency [56]. Antiproliferative and antioxidant activities of *F. cretica* have also been reported in both in vitro and in vivo studies, along with physicochemical characterization, efficacy evaluation, and preliminary safety assessment [4]. Simultaneous measurement of antiproliferative and antioxidant activity may be methodologically valuable because it highlights the dual behavior of plant-derived antioxidants: cytoprotective in normal cells while exerting pro-oxidant effects in cancer cells.

The anti-inflammatory and cytotoxic activities of *F. cretica* were further investigated by Mohamed and colleagues using metabolic profiling, in vitro cytotoxicity assays, and in silico target prediction from Egyptian desert populations of the plant [27]. The extract demonstrated anticancer activity, and its biological profile was attributed largely to anti-inflammatory mechanisms. Pharmacologically, there is a strong link between anti-inflammatory and anticancer activity because chronic inflammation contributes to tumor cell survival through NF-κB-mediated survival signaling. Consequently, agents that inhibit NF-κB signaling may exert both anti-inflammatory and anticancer effects. In silico tools were used to identify potential molecular targets. However, these findings should be considered hypothesis-generating and require experimental validation. The crude methanolic extract of *F. cretica* has also been shown to modulate the expression of TGF-β receptors and SMAD genes in human cancer cell lines, suggesting potential interference with TGF-β signaling pathways associated with tumor invasion and metastasis in advanced cancers [57]. The relatively high concentrations of phenolics (4.38–9.62 mg GAE/g), flavonoids (5.11–7.16 mg/g), tannins (6.66–12.44 mg/g), alkaloids (3.32–4.43 mg/g), and saponins (5.87–8.14 mg/g), together with measurable antioxidant activity (1.40 mg/g), indicate a substantial capacity to maintain redox homeostasis [26]. These phytochemicals may act synergistically through ROS scavenging, activation of endogenous antioxidant defense systems, suppression of oxidative stress-induced inflammatory pathways, and protection of cellular biomolecules and mitochondrial function from oxidative damage.

**Figure 4:**
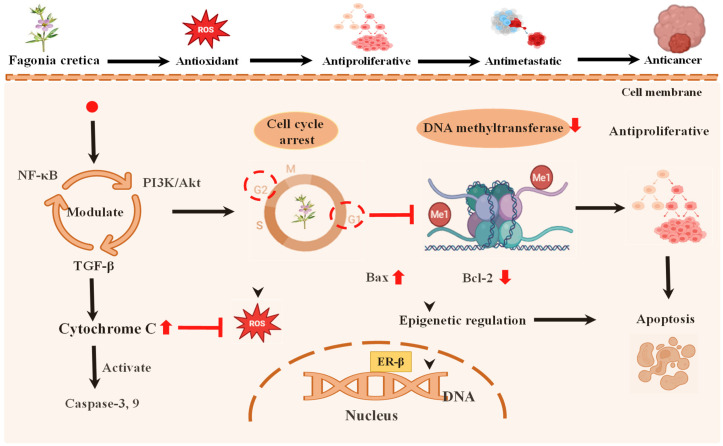
Redox homeostasis-mediated anticancer mechanisms of bioactive compounds of *F. cretica* through regulation of oxidative stress, cell cycle arrest, epigenetic modulation, and apoptosis. The figure illustrates the anticancer mechanisms of these bioactive compounds through modulation of redox homeostasis, oxidative stress, apoptotic signaling, and epigenetic regulation. Bioactive compounds regulate intracellular reactive oxygen species (ROS) levels and suppress oxidative stress-mediated oncogenic pathways, including nuclear factor kappa B (NF-κB), phosphoinositide 3-kinase/protein kinase B (PI3K/Akt), and transforming growth factor-beta (TGF-β) signaling, thereby reducing inflammation, proliferation, and tumor progression. These compounds arrest the cell cycle at various checkpoints, including the gap 1 (G_1_), synthesis (S), gap 2 (G_2_), and mitotic (M) phases, thereby preventing the uncontrolled proliferation of cancer cells. At the same time, suppression of DNA (deoxyribonucleic acid) methyltransferase activity contributes to epigenetic regulation and the reactivation of tumor suppressor genes. The mitochondrial apoptotic pathway is triggered by an increase in Bcl-2-associated X protein (Bax) and a decrease in B-cell lymphoma 2 (Bcl-2) expression, resulting in the release of cytochrome c and activation of cysteine-aspartic protease-3 (caspase-3) and cysteine-aspartic protease-9 (caspase-9), which ultimately induce apoptosis. Additionally, transcriptional regulation mediated by estrogen receptor beta (ER-β) further contributes to the inhibition of cancer progression.

The use of *F. cretica* water extracts for nanoparticle synthesis has also been explored as a strategy to improve anticancer drug delivery. Kiani et al. synthesized zinc oxide nanoparticles using extracts from *F. cretica* and demonstrated anticancer activity, which they attributed to the combined pro-oxidant effects of zinc oxide nanoparticles and the phytochemical coating derived from the plant extract [58]. Studies on Fagonia-mediated nanoparticles further indicate that green-synthesized nanoparticles exhibit consistent anticancer activity in cell-based assays [59,60]. Although these findings are promising, nanoparticle-based approaches introduce additional variables, such as particle size, surface charge, and stability, that must be carefully characterized before biological effects can be confidently attributed to the plant-derived compounds rather than the nanoparticle carrier itself. The major redox-linked anticancer mechanisms discussed in this section are summarized in Figure 4.

Water-extract fractions and acid hydrolysates from Libyan populations of *F. cretica* showed cytotoxic activity against cancer cell lines, and saponin glycosides were proposed as possible contributors to this activity. However, because the active preparations were fractionated mixtures, the observed cytotoxicity should be attributed to the tested fractions rather than to a single compound class. Separately, quinovic acid glycosides may be discussed as triterpenoid glycosides, whereas betulin and taraxerol are free triterpenes, diosgenin is a steroidal sapogenin, and harmine is an alkaloid. These metabolites should therefore not be described collectively as saponins [11]. Quinovic acid glycosides, betulin, diosgenin, taraxerol, and harmine have been associated with anticancer activities through the modulation of multiple molecular pathways involved in tumor progression [29,30]. These compounds can induce apoptosis, inhibit uncontrolled cell proliferation, and suppress oxidative stress-induced DNA damage that contributes to carcinogenesis. Furthermore, their antioxidant and anti-inflammatory properties reduce ROS-mediated activation of NF-κB and other pro-survival signaling pathways. The combined effects on cell cycle regulation, mitochondrial function, and inflammatory mediators may contribute to the prevention and suppression of cancer development.

The available evidence suggests that extracts and compounds from *F. cretica* possess cytotoxic and antiproliferative properties mediated by the induction of oxidative stress, activation of apoptosis, epigenetic modifications, and disruption of inflammatory and growth factor signaling pathways [27,55]. These mechanisms are biologically plausible and consistent with the redox pharmacology of flavonoid-rich plant extracts. However, it is important to note that the current evidence base has several limitations. Most studies rely on crude or partially characterized extracts rather than isolated compounds. The observed biological effects cannot be attributed to a single molecule. Additionally, in vitro doses and concentrations often do not correspond to tissue concentrations achievable in vivo according to pharmacokinetic constraints. There are also inconsistencies in the reporting of positive and negative controls, and many proposed mechanisms have not yet been validated in vivo. These limitations do not invalidate the reported findings, but they highlight the need for further research. Future investigations should include compound-level mechanistic studies, comprehensive in vivo pharmacological assessments using validated redox biomarkers, and well-designed clinical investigations before the anticancer potential of *F. cretica* can be responsibly translated into therapeutic recommendations (Table 2).

### 5.4. Hepatoprotective Mechanism

Hepatic diseases (including NAFLD, NASH, cirrhosis, and hepatocellular carcinoma (HCC)) represent a model system that mechanistically demonstrates how chronic redox imbalance contributes to progressive fibrogenesis and malignant transformation. The physiological ROS load of hepatocytes is among the highest of all cell types, as ROS are produced simultaneously through CYP2E1 activity, peroxisomal beta-oxidation, mitochondrial respiratory leakage, and Kupffer cell NADPH oxidase [69,70,71]. Initially, caloric excess leads to steatosis, a process in which ROS generated by fatty acid peroxidation activate NF-κB signaling, resulting in the generation of 4-HNE and MDA. These reactive products form adducts with hepatic proteins and DNA and activate hepatic stellate cells, thereby promoting fibrogenesis [72]. A second hit involving ROS-mediated NF-κB activation further increases 4-HNE and MDA production, which subsequently drives stellate cell activation toward myofibroblast differentiation and collagen deposition. This process promotes disease progression from steatohepatitis to fibrosis and cirrhosis and eventually to HCC [73]. CYP2E1-mediated ROS production selectively depletes mitochondrial GSH, while acetaldehyde–protein adducts enhance inflammatory cascades in alcohol-related liver disease [74]. The stage-dependent nature of redox-based therapies is well illustrated by the Nrf2 pathway. Although strong hepatoprotective effects are observed in early NAFLD, the same Nrf2 activation in established HCC can result in tumor antioxidant shielding and therapy resistance. This highlights the need for stage-specific targeting of the Nrf2 pathway, such as the use of Nrf2 activators (e.g., sulforaphane and bardoxolone methyl) in early metabolic disease, compartment-specific antioxidants (e.g., MitoQ), and pro-oxidant ferroptosis-based strategies in advanced malignancy.

*F. cretica* exhibits hepatoprotective effects mainly through antioxidant, anti-inflammatory, and anti-apoptotic mechanisms. Its bioactive compounds activate the Nrf2 pathway, which enhances antioxidant enzymes (SOD, CAT, and GPx) and reduces oxidative stress by lowering ROS levels and lipid peroxidation in liver cells [16]. At the same time, it inhibits the NF-κB signaling pathway, leading to decreased release of pro-inflammatory cytokines such as TNF-α, IL-1β, and IL-6, thereby reducing hepatic inflammation. Additionally, it protects hepatocytes by modulating the mitochondrial apoptosis pathway (↑ Bcl-2, ↓ Bax), preventing cytochrome c release and caspase activation. It may also support liver regeneration by activating the PI3K/Akt survival pathway [17]. Furthermore, *F. cretica* helps maintain hepatocyte membrane integrity by preventing toxin-induced damage caused by drugs, alcohol, or oxidative stress. It also reduces hepatic lipid accumulation, thereby improving liver metabolic function and reducing the risk of steatosis [75]. The combined action of these pathways promotes detoxification, cellular repair, and the restoration of normal liver architecture. Overall, its hepatoprotective effects result from the combined reduction in oxidative damage, suppression of inflammation, and prevention of hepatocyte death [59].

Recent studies have highlighted the considerable therapeutic potential of *F. cretica* in various disease models due to its abundant phytochemical composition and diverse biological activities. For example, PEGylated nanocomposites containing *F. cretica* methanolic extract and silymarin were reported in Scientific Reports to alleviate oxidative stress, reduce ROS, and decrease DNA damage in CCl_4_-induced hepatotoxic rats, demonstrating stronger hepatoprotective effects than conventional silymarin treatment [43]. The presence of quinovic acid derivatives, β-amyrin acetate, taraxerol, betulin, stigmasterol, and campesterol suggests substantial hepatoprotective potential through attenuation of oxidative stress and inflammation. These bioactive compounds enhance antioxidant defense systems, reduce ROS generation, and inhibit lipid peroxidation in hepatic tissues. In addition, triterpenoids and phytosterols suppress NF-κB-mediated inflammatory signaling and protect hepatocytes from oxidative injury. Their ability to preserve mitochondrial integrity and stabilize cellular membranes further contributes to the maintenance of liver function and prevention of hepatic damage [28,30]. Moreover, both in vitro and in vivo studies have demonstrated the antiproliferative and antioxidant effects of *F. cretica*, including tumor regression, increased body weight, and no significant adverse effects on liver or kidney function in cancer models [16]. The cytotoxic and anti-inflammatory actions of *F. cretica* have also been associated with inhibition of COX enzymes, induction of apoptotic proteins (such as caspase-9), and strong binding affinity of its metabolites to cancer-related molecular targets [27]. In addition, biosynthesized selenium nanoparticles (SeNPs) derived from *F. cretica* have demonstrated promising antidiabetic properties by lowering blood glucose levels, inhibiting α-glucosidase and α-amylase, and improving biochemical parameters in diabetic mice [46].

### 5.5. Antidiabetic Mechanisms

Insulin resistance and type 2 diabetes result from bidirectional mitochondrial redox dysfunction, in which the signaling molecule H_2_O_2_ can be protective at physiological levels but becomes destructive when produced in excess for prolonged periods [76]. Inactivation of protein tyrosine phosphatase 1B (PTP1B) by moderate levels of H_2_O_2_ is a physiologically documented redox amplification mechanism that leads to increased phosphorylation of the insulin receptor and IRS-1 [77]. However, during chronic overnutrition, excessive substrate flux through mitochondrial Complexes I and III overwhelms MnSOD and peroxiredoxin-3, resulting in the accumulation of cytoplasmic superoxide. This process drives three compounding consequences: first, diacylglycerol and ceramide accumulation activates PKCθ and PKCε, triggering phosphorylation of IRS-1 at inhibitory serine residues and thereby reducing insulin signaling. Second, NF-κB stimulates adipose tissue secretion of paracrine cytokines such as TNF-α and IL-6, which inhibit IRS-1 signaling in skeletal muscle and liver. Third, non-enzymatic glycation of proteins and lipids generates advanced glycation end products (AGE), which bind to the receptor for AGE (RAGE), maintaining NF-κB activation in a pathological feed-forward loop [78]. Unlike many other cell types, pancreatic β-cells possess relatively low levels of catalase and glutathione peroxidase. Consequently, glucose-induced endoplasmic reticulum stress and mitochondrial ROS production lead to irreversible β-cell apoptosis, ultimately resulting in the transition to overt diabetes. Plasma F_2_-isoprostanes, urinary 8-OHdG, the erythrocyte GSH/GSSG ratio, and HbA1c as an integrated measure of glycemic control and protein glycation are recognized as clinically validated redox biomarkers [79].

*F. cretica* exhibits significant potential in the management of diabetes through a multifaceted mechanism involving glucose regulation, insulin sensitization, and metabolic modulation. Its bioactive compounds, including flavonoids, saponins, and polyphenols, help maintain blood glucose balance by enhancing insulin release from pancreatic β-cells and improving insulin sensitivity in peripheral tissues [46]. A key mechanism involves activation of the AMPK pathway, which facilitates glucose uptake in muscle and adipose tissues via GLUT4 translocation while simultaneously inhibiting hepatic gluconeogenesis, thereby lowering blood glucose levels [80]. Furthermore, *F. cretica* mitigates diabetes-associated oxidative stress by increasing antioxidant enzyme activity (SOD, catalase, and glutathione) and reducing ROS, thereby protecting pancreatic cells from oxidative damage. It also suppresses inflammatory signaling pathways, including NF-κB, thereby reducing chronic inflammation associated with insulin resistance [81]. In addition, the plant inhibits digestive enzymes such as α-amylase and α-glucosidase, resulting in slower carbohydrate digestion and reduced postprandial glucose spikes. Components of the saponin fraction and β-sitosterol from *F. cretica* are predicted to modulate the PI3K/Akt/mTOR signaling pathway through PTEN stabilization, direct inhibition of Akt kinase, and activation of AMPK. AMPK functions as a metabolic sensor that antagonizes mTORC1 activity during energy-deficient states. Importantly, inhibition of mTOR promotes autophagy, a cellular housekeeping process that removes oxidatively damaged organelles (mitophagy) and protein aggregates, thereby reducing the overall oxidative stress burden [26]. In summary, the antidiabetic effects of *F. cretica* arise from a combination of mechanisms involving insulin secretion, glucose uptake, digestive enzyme inhibition, antioxidant protection, and anti-inflammatory regulation [82].

**Figure 5:**
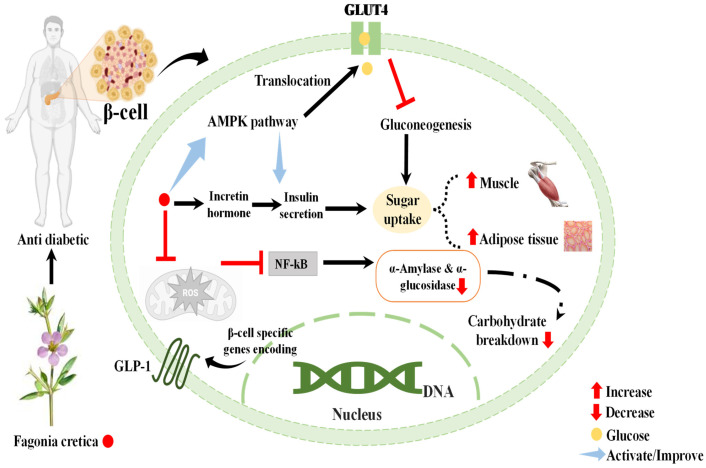
Molecular mechanisms underlying the antidiabetic and anti-inflammatory effects of *F. cretica* through modulation of ROS, glucose uptake, and NF-κB signaling. The bioactive compounds of *F. cretica* enhance glucose uptake and improve insulin sensitivity while suppressing ROS-mediated inflammatory pathways in pancreatic β-cells and peripheral tissues. The extract reduces the production of ROS and decreases NF-κB signaling, thereby lowering inflammation and cellular damage. Furthermore, inhibition of α-amylase and α-glucosidase activities reduces carbohydrate digestion and glucose absorption, resulting in improved glycemic control. Collectively, these mechanisms support β-cell protection, maintenance of cellular DNA integrity, and overall metabolic homeostasis.

*F. cretica* has shown considerable potential for diabetes management through several complementary mechanisms, as demonstrated by in vitro, in vivo, and molecular studies. The plant effectively inhibits key enzymes involved in carbohydrate digestion, particularly α-glucosidase (IC_50_ = 4.62 µg/mL), thereby reducing postprandial glucose absorption. It also inhibits α-amylase, especially in nanoparticle-based formulations [46,83]. Furthermore, *F. cretica* enhances incretin-mediated insulin secretion by activating the TGR5 receptor and promoting the release of glucagon-like peptide-1 (GLP-1), while simultaneously inhibiting dipeptidyl peptidase-4 (DPP-4), thereby prolonging incretin activity [30,84]. In vivo studies using streptozotocin-induced diabetic models have shown that administration of *F. cretica* methanolic extract at doses of 250–500 mg/kg significantly reduced blood glucose levels (by up to 45%) and protected pancreatic β-cells from damage [83]. Additionally, biosynthesized nanoparticles (AgNPs and SeNPs) enhanced antidiabetic efficacy by improving lipid profiles, liver and kidney function, and reducing oxidative stress, highlighting their role in systemic metabolic regulation [46]. The presence of bioactive compounds such as flavonoids (quercetin and kaempferol), triterpenoids, and saponins further supports the plant’s multitarget antidiabetic activity, including modulation of drug-metabolizing enzymes (CYP3A4 and UGT2B7), which may influence therapeutic outcomes in diabetic patients [31]. The proposed antidiabetic and anti-inflammatory signaling mechanisms are summarized in Figure 5.

### 5.6. Antimicrobial Activities

*F. cretica* water extract demonstrates antimicrobial properties primarily due to its rich phytochemical composition, which includes flavonoids, phenolics, saponins, tannins, and alkaloids. These compounds inhibit the growth of bacteria and fungi through multiple mechanisms. The antimicrobial action begins with disruption of the microbial cell wall and membrane integrity. Saponins and tannins interact with membrane lipids and proteins, increasing membrane permeability and causing leakage of essential intracellular components. This leads to the loss of ions and metabolites and ultimately results in microbial cell death [85]. Flavonoids and phenolic compounds also contribute to antimicrobial activity by inhibiting nucleic acid synthesis through interference with DNA gyrase and topoisomerase, thereby blocking DNA replication and protein synthesis in microbes. In addition, these bioactive compounds interfere with microbial energy metabolism by inhibiting key enzymatic systems involved in ATP production [86]. At the molecular signaling level, oxidative damage induced by plant metabolites increases ROS levels within microbial cells, resulting in oxidative stress–mediated damage to proteins, lipids, and DNA [17].

Recent research highlights the considerable antimicrobial, antioxidant, and biomedical potential of *F. cretica*, particularly in combating multidrug-resistant (MDR) pathogens and in the development of nanoparticle-based therapeutics. A clinical study investigating MDR biofilm-forming isolates from diabetic foot ulcers reported that nanoparticles synthesized using *F. cretica* and *Withania coagulans* exhibited strong antimicrobial activity against resistant bacterial and fungal isolates, suggesting potential applications in the treatment of chronic wounds [87]. Similarly, green-synthesized zinc oxide nanoparticles (ZnO NPs) produced using *F. cretica* water extract demonstrated significant antibacterial activity against both Gram-positive and Gram-negative bacteria, along with notable antioxidant capacity, indicating their environmental compatibility and biomedical relevance [88]. Phytochemical investigations have also revealed that *F. cretica* contains several bioactive compounds, including gallic acid, quinic acid, liquiritigenin, and rosmarinic acid, which contribute to its activity against MDR gastrointestinal pathogens [33]. Furthermore, nanoparticle synthesis using plant extracts has been shown to enhance biological activities, including antioxidant, antimicrobial, cytotoxic, and antidiabetic effects, highlighting the potential of medicinal plants as valuable sources of functional nanomaterials [59].

### 5.7. Antidepressant Activity

*F. cretica* has been investigated for its potential antidepressant effects, primarily due to its high content of flavonoids, phenolics, and saponins, which may influence central nervous system signaling and oxidative balance. The plant’s antidepressant activity is mainly associated with the modulation of monoaminergic neurotransmission and neuroinflammatory pathways. Bioactive compounds present in *F. cretica* are believed to increase the availability of key neurotransmitters such as serotonin (5-HT), dopamine, and norepinephrine, either by enhancing their synthesis or by inhibiting their reuptake, thereby improving mood and reducing depressive symptoms [89]. Additionally, *F. cretica* may reduce neuroinflammation by inhibiting the NF-κB signaling pathway, which leads to decreased production of pro-inflammatory cytokines such as TNF-α and IL-1β, both of which are closely associated with the pathophysiology of depression. Concurrently, its strong antioxidant properties may mitigate oxidative stress in brain tissues by enhancing endogenous antioxidant enzymes (SOD, CAT, and GPx) and reducing ROS, thereby protecting neuronal cells from damage [58].

One study evaluating the pharmacological effects of five indigenous Pakistani medicinal plants, including *F. cretica*, using in vivo rat models reported significant biological activities. Among the plants tested, *F. cretica* demonstrated a pronounced anticoagulant effect by markedly prolonging clotting time, suggesting potential blood-thinning properties. Additionally, it contributed to the overall anti-inflammatory outcomes of the study by exhibiting moderate inhibition of inflammation in dose-dependent assays (Table 3).

These findings indicate that *F. cretica* contains bioactive phytochemicals such as flavonoids, tannins, and saponins that may modulate pain, inflammation, and coagulation pathways. The study therefore highlights the potential of *F. cretica* as a multifunctional medicinal plant warranting further pharmacological and therapeutic investigation [51]. Overall, the experimental evidence supporting the redox-related pharmacological activity of *F. cretica* is derived mainly from in vitro assays, animal models, nanoparticle-based formulations, and computational studies, as shown in Table 2 and Table 3. Antioxidant evidence includes radical-scavenging assays, reduced ROS generation, decreased lipid peroxidation, and increased antioxidant enzyme activity. Anti-inflammatory evidence includes inhibition of COX-1/COX-2, nitric oxide production, NF-κB activation, and pro-inflammatory cytokine expression. Anticancer evidence includes cytotoxicity against HepG2, MCF-7, Caco-2, HCT-116, and PC3 cells, with reported mechanisms involving apoptosis, topoisomerase inhibition, oxidative stress induction, DNMT1 downregulation, ER-β activation, and p53/FOXO3a signaling. Antidiabetic evidence includes α-amylase and α-glucosidase inhibition, improved glucose control in diabetic models, β-cell protection, and reduced oxidative stress.

## 6. Limitations and Future Directions

Despite the growing pharmacological interest in *F. cretica*, a fundamental limitation of the existing literature lies in the incomplete and inconsistent phytochemical characterization of its bioactive constituents across different geographical accessions, plant parts, and extraction methodologies. Most studies have utilized either crude extracts or partially fractionated preparations, making it difficult to identify the specific molecular entities responsible for the observed antioxidant, anti-inflammatory, or cytotoxic effects, particularly those containing defined redox-active pharmacophores. Although flavonoids, saponins, alkaloids, and iridoids have been generally identified, comprehensive structure–activity relationship (SAR) analyses linking individual compounds to their ability to influence the Nrf2–Keap1 axis, NF-κB signaling, or the GSH/GSSG redox couple are largely absent from the current literature. This limitation is particularly important given the growing recognition that antioxidant bioactivity is not uniform but highly context-dependent and influenced by factors such as dosage, cellular redox environment, and the specific oxidative stress pathway involved. Without detailed mechanistic insights at the compound level, it is difficult to determine whether the biological effects observed in *F. cretica* aqueous extracts result from true redox modulation, nonspecific cytotoxicity, hormetic low-dose pro-oxidant activation of adaptive pathways, or simple radical scavenging without meaningful intracellular effects. Moreover, the lack of validated pharmacokinetic data—including oral bioavailability, metabolic transformation, tissue distribution, and blood–brain barrier permeability of major bioactive molecules—means that in vitro antioxidant activity assays, commonly performed using DPPH, ABTS, and FRAP methods, cannot be reliably extrapolated to in vivo or clinical settings. This represents a critical gap that significantly limits the interpretive value of published findings.

Another major methodological limitation in current research on *F. cretica* is the excessive reliance on simple chemical antioxidant assays such as DPPH, ABTS, and FRAP. These assays measure radical-scavenging activity under artificial, non-physiological conditions and do not provide meaningful insight into intracellular redox regulation. Consequently, they fail to capture the potential of bioactive compounds to influence the Nrf2–Keap1 pathway, regulate GSH/GSSG ratios, or activate enzymatic antioxidant defenses such as SOD, catalase, and glutathione peroxidase. Furthermore, the absence of validated intracellular redox biomarkers, particularly 8-OHdG as a marker of oxidative DNA damage, F_2_-isoprostanes as indicators of lipid peroxidation, and protein carbonyls as markers of oxidative protein modification, means that current studies cannot determine whether the reported biological effects represent true restoration of redox homeostasis or nonspecific chemical reactivity lacking intracellular relevance. Additionally, most in vitro experiments are conducted in single cancer cell lines under standard atmospheric oxygen levels that do not reflect the hypoxic, inflammatory, or metabolically dysregulated microenvironments present in diseased tissues, thereby limiting the biological validity and translational applicability of the reported results.

A major obstacle to the clinical application of *F. cretica* research is the near absence of comprehensive in vivo studies and human clinical data evaluating its safety, efficacy, and redox-specific pharmacodynamics in major diseases such as cancer, cardiovascular disease, type 2 diabetes, and liver disorders. Existing animal studies are further limited by the use of non-standardized extract preparations, the absence of pharmacokinetic analyses, and the failure to consider the context-dependent effects of antioxidant interventions. This issue is particularly critical in oncology, where antioxidants may shield tumor cells from oxidative damage, promote metastasis, and reduce the effectiveness of pro-oxidant chemotherapeutic agents. In addition, the toxicological profile of *F. cretica* bioactive compounds remains poorly characterized, with limited data available regarding hepatotoxicity, genotoxicity, and potential interactions with cytochrome P450 enzymes that could influence the metabolism of co-administered drugs or endogenous redox-sensitive cofactors such as glutathione and tetrahydrobiopterin. Without systematic investigation using validated redox biomarker panels, disease stage-specific animal models, and well-designed clinical trials, the therapeutic application of *F. cretica* within a precision redox medicine framework remains premature. Consequently, the risk of adverse outcomes from inappropriate antioxidant use—particularly in patients with active cancer or those undergoing oxidative stress-based therapies—cannot currently be excluded. The translational potential of *F. cretica*-derived compounds remains promising but preliminary. Current evidence supports further investigation of standardized extracts or isolated marker compounds as redox-modulating candidates in diseases characterized by oxidative stress and chronic inflammation. However, clinical use cannot yet be recommended because standardized formulations, dose–response data, pharmacokinetic profiles, bioavailability, toxicity, herb–drug interaction studies, and controlled clinical trials are still insufficient. Translational development should therefore prioritize quality-controlled extracts, validated redox biomarkers, disease-stage-specific models, and comprehensive safety evaluation before progressing toward clinical application.

## 7. Conclusions

In conclusion, current evidence suggests that *F. cretica* L. is a phytochemically diverse medicinal plant with potential biological activities mediated through redox-sensitive signaling pathways. Experimental studies indicate that its bioactive constituents may influence oxidative stress responses, inflammatory signaling, metabolic regulation, and cancer cell survival pathways. However, most available data originate from in vitro experiments or limited animal studies using non-standardized extracts. Future investigations should therefore focus on the isolation of active compounds, standardized extraction procedures, pharmacokinetic characterization, and validation of redox-specific biomarkers in well-designed in vivo models and clinical studies. Such efforts will be essential to determine whether the promising pharmacological properties attributed to *F. cretica* can be translated into safe and effective therapeutic strategies.

## Figures and Tables

**Table 1:** Nutritional composition, mineral profile, phytochemical classes, and major bioactive constituents of *Fagonia cretica* L. ^1^.

Category	Composition	Reported Biological Activities	References
Nutritional composition	Moisture (10.41%), ash (9.22 ± 0.77%), crude fiber (14.57%), lipids (4.63%), protein (9.34%), total carbohydrates (622.4 mg/g), total soluble sugars (29.68 mg/g), polysaccharides (133.47 mg/g), glucose (1.13 mg/g), sucrose (9.67 mg/g), energy value (327.99 kcal/100 g)	Supports cellular metabolism and physiological energy requirements; contributes to redox balance through nutritional antioxidant support (general nutritional function only, not a direct pharmacological claim)	[26]
Mineral composition	Na (13.09 mg/g), K (12.09 mg/g), Ca (10.69 mg/g), Mg (7.19 mg/g), Fe (1.13 mg/g), Cu (0.31 mg/g), Mn (0.09 mg/g), Zn (0.07 mg/g)	Functions as enzymatic cofactors and supports antioxidant enzyme systems (e.g., SOD, catalase)	[26]
Total phenolics and flavonoids	Total phenolic content (2.4 mg GAE/g), flavonoid content (0.18 mg GAE/g)	Associated with radical-scavenging capacity and reduction in oxidative stress markers in vitro	[27]
Phenolic compounds (phenolic acids and flavonoids)	Gallic acid, quercetin, vanillic acid, benzoic acid, m-coumaric acid, sinapic acid, cinnamic acid, rosmarinic acid (0.97–11.21 ppm range across different extracts)	Strong antioxidant and anti-inflammatory potential; free radical scavenging; NF-κB modulation and redox regulation (literature-supported)	[31,32]
Flavonoid fraction (quantified content)	Total flavonoids (0.18 mg GAE/g) and quercetin (7.82–8.28 ppm in extracts)	Contributes to antioxidant activity and inhibition of oxidative stress–induced cellular damage	[27,31]
Saponins (triterpenoid glycosides)	Oleanane-type triterpenoid saponins (glycosylated quinovic/oleanolic acid derivatives; 5.87–8.14 mg/g)	Reported membrane interaction, antimicrobial and cytotoxic potential at compound-class level	[27]
Free triterpenes and sterols	Quinovic acid derivatives, β-amyrin acetate, taraxerol, oleanolic aldehyde acetate, betulin, diosgenin, cryptogenin, stigmasterol, campesterol, lanosterol	Antioxidant, membrane-stabilizing, and anti-lipid peroxidation activities; potential involvement in redox signaling (e.g., Nrf2-associated pathways)	[28,29,30]
Fatty acids and lipid-derived compounds	Linoleic acid, triacontanoic acid	Contribute to membrane integrity and lipid metabolism; reported anti-lipid peroxidation potential	[28,29,30]
Alkaloids and nitrogenous compounds	Harmine (part of isolated phytochemical fraction)	Reported bioactivity includes antioxidant and neuroactive potential based on literature evidence	[28,29,30]
Low-molecular-weight metabolites (aqueous extract profile)	Succinic acid, quinic acid, cyclo(L-Leu-L-Pro), liquiritigenin, cerebronic acid	Reported antioxidant, anti-inflammatory, hepatoprotective, cardioprotective, and anti-fibrotic activities (compound-based evidence)	[32]

^1^ Quantitative values are presented as reported in the cited studies. Percentage values refer to g/100 g of plant material, mg/g values refer to milligrams per gram of plant material, and ppm values refer to concentrations detected in the analyzed extract by HPLC. The basis of expression, such as dry weight, fresh weight, or extract weight, is specified where available; NR indicates that the original study did not report the basis of expression.

**Table 2:** Preclinical anticancer evidence for *Fagonia cretica* L. extracts, fractions, and nanoformulations.

Type of Drug	Type of Study	Type of Cancer	Cancer Cell Line	Mechanism	Reference
Ethanolic extract, hexane extract	In vitro, In silico	Liver cancer	HepG2	Cytotoxic activity, apoptotic induction, inhibition of TNF-α and TGF-β	[17]
Alcoholic extract	metabolic, in vitro, and in silico profiling	Liver cancer breast cancer, intestinal cancer	HepG2, MCF-7, Caco-2	COX-2, COX-1, and nitric oxide inhibition, impact on apoptotic markers (topoisomerase I and caspase 9 enzymes), effect on anti-inflammatory (COX-2 and COX-1) and cytotoxicity (topoisomerases I, IIα, and IIβ)	[27]
Aqueous extract	In vitro, in vivo	Breast cancer	MCF-7	Performed scavenging activity through MTT assay, regress tumor, possesses anticancer, antioxidant, and cytotoxic properties	[4]
Aqueous extract	In vitro	Breast cancer	MCF-7, MDA-MB-231	*F. cretica* extract induces DNA damage, causing G0/G1 cell cycle arrest and apoptosis via p53 (p21, BAX). p53-deficient cells, activates FOXO3a, leading to growth inhibition and cell death.	[61]
Methanolic extract, aqueous extract	In vitro	Breast cancer, hepatocellular carcinoma, laryngeal carcinoma	MCF-7, Hep-2, HUH-7, HCEC	TRAIL-mediated extrinsic apoptotic pathway—upregulation of TRAIL, DR4, DR5, FADD, and TP53 genes, caspase-dependent apoptosis via death receptor signaling, no change in cFLAR (anti-apoptotic inhibitor remained suppressed)	[57]
Ethanolic extract	In vitro, in silico	Colorectal prostate	HCT-116, PC3	Induces antiproliferative and antiapoptotic effects through increased ROS production	[55]
Methanolic extract, aqueous extract	In vitro	Breast, lung, oral	MCF-7, A549, KB-3-1, L929 (normal, non-cancerous control)	Apoptosis induction via: ROS-mediated oxidative stress, DNA damage, cell necrosis	[62]
Methanolic extract	In vitro	Hepatocellular carcinoma, breast cancer	HUH, MCF-7	Antiproliferative effect, downregulated TGFβR1, TGFβR2, TGFβR3 and SMAD3 expression, decreased SMURF1/2 expression	[57]
Methanolic extract	In vitro	Breast cancer	MCF-7	Apoptosis induction via upregulation of Caspase-1, -3, -7, and -9, down regulation of Wnt-3a and β-catenin	[63]
Aqueous extract	In vitro	Liver cancer	HepG-2	ROS-mediated cytotoxicity inducing oxidative stress, cell membrane damage	[64]
Ethanolic extract	In vivo	Breast cancer	MCF-7, MDA-MB-231	Decreases p-53 and increases FOXO3a, inhibits upregulation of IkBα and COX-1 genes in liver	[65]
Ethanolic extract, chloroformic extract	In vitro, in vivo	Breast cancer	MCF-7, MDA-MB-231	Induces cell cycle arrest and apoptosis, increases WBC count, increases platelet count	[66]
Chloroform fraction	In vitro	Breast cancer	MCF-7, MDA-MB-231	Induces ROS-mediated apoptosis, loss of mitochondrial membrane potential, activation of Caspase-3	[67]
Crude extract	In vitro	Breast cancer, cervical cancer	MCF-7, MDA-MB-231 HeLa, MCF-10A	Cell cycle arrest, apoptosis, activation of p53, Bax, p21	[68]

Abbreviations used: A549, human lung adenocarcinoma cell line; BAX, Bcl-2-associated X protein; Caco-2, human colorectal adenocarcinoma cell line; cFLAR, CASP8 and FADD-like apoptosis regulator; COX-1, cyclooxygenase-1; COX-2, cyclooxygenase-2; DNA, deoxyribonucleic acid; DR4, death receptor 4 (TNFRSF10A); DR5, death receptor 5 (TNFRSF10B); FADD, Fas-associated death domain protein; FOXO3a, forkhead box O3a; G0/G1, gap 0/gap 1 phase of the cell cycle; HCEC, human corneal epithelial cells; HCT-116, human colorectal carcinoma cell line; HeLa, human cervical adenocarcinoma cell line; Hep-2, human laryngeal carcinoma cell line; HepG2, human hepatocellular carcinoma cell line; HUH-7, human hepatocellular carcinoma cell line; IκBα, inhibitor of nuclear factor kappa-B alpha; KB-3-1, human oral epidermoid carcinoma cell line; L929, mouse fibroblast cell line; MCF-10A, human non-tumorigenic mammary epithelial cell line; MCF-7, human breast adenocarcinoma cell line; MDA-MB-231, human triple-negative breast cancer cell line; MTT, 3-(4,5-dimethylthiazol-2-yl)-2,5-diphenyltetrazolium bromide assay; NO, nitric oxide; p21, cyclin-dependent kinase inhibitor 1A (CDKN1A); p53, tumor protein p53; PC3, human prostate adenocarcinoma cell line; ROS, reactive oxygen species; SMAD3, mothers against decapentaplegic homolog 3; SMURF1, SMAD-specific E3 ubiquitin protein ligase 1; SMURF2, SMAD-specific E3 ubiquitin protein ligase 2; TGF-β, transforming growth factor-beta; TGFβR1, transforming growth factor-beta receptor 1; TGFβR2, transforming growth factor-beta receptor 2; TGFβR3, transforming growth factor-beta receptor 3; TNF-α, tumor necrosis factor-alpha; TRAIL, tumor necrosis factor-related apoptosis-inducing ligand; WBC, white blood cells; Wnt-3a, wingless-related integration site 3a protein.

**Table 3:** Summary of antidiabetic, hepatoprotective, antimicrobial, and related pharmacological evidence for *F. cretica*.

	Model	Formulation/ Intervention	Dose	Key Findings	Mechanism	Study
Anti-diabetic and hepatoprotective	In vitro	Leaves extract	62–1000 µg/mL	↓ Glucose (105 mg/dL), strong enzyme inhibition	α-amylase and α-glucosidase inhibition, antioxidant	[46]
	STZ-induced diabetic mice	Leaves AgNPs	5, 10, 15, and 20 mg/kg			
	In vitro	Unfractionated extract; solvent NS plant part NS	Not specified	DPP-4 inhibition (IC_50_ 38.1 µg/mL)	Incretin preservation	[30]
	Cell line	Unfractionated extract; solvent NS; plant part NS.	125 and 250 µg/mL	↑ GLP-1 secretion	TGR5 activation	[84]
	STZ rats	Extract of aerial parts	500 mg/kg	Modulated drug-metabolizing enzymes	CYP3A4 ↓, UGT2B7 ↑	[31]
	In vitro	Solvent extract of aerial parts	Not specified	52.6% antidiabetic activity	Antioxidant, protein kinase inhibition	[58]
	In vitro	Alcoholic extract of dried aerial parts	Not specified	Strong antioxidant and anti-inflammatory	Indirect insulin sensitivity improvement	[27]
	In vitro	Leaf extract	Not specified	Enhanced hepatocyte survival and tissue regeneration	Activation of PI3K/Akt pathway, modulation of mitochondrial apoptosis (↑ Bcl-2, ↓ Bax, ↓ caspase activation)	[17]
	Animal model, clinical study	Dietary intervention pharmacological agents	Not specified	Imbalance of homeostasis, mitochondrial oxidation increases	Increased expression and translocation of transporters (like CD36) from the cytoplasm to the plasma membrane	[75]
	In vivo liver injury models	Extracts of *Fagonia* species	Not specified	Reduced inflammation, improved liver function and membrane integrity	Inhibition of NF-κB pathway → ↓ TNF-α, ↓ IL-1β, ↓ IL-6, reduced lipid accumulation and hepatocyte damage	[59]
Antioxidant	CCl_4_-induced hepatotoxic rats	Plant extract	1500 µg/kg	Reduced ROS, TBARS, DNA damage, increased SOD, CAT, POD	Antioxidant activity, hepatoprotection	[43]
	Hyperuricemic mice and cell lines	Unfractionated extract; solvent NS; plant part NS.	*Withania coagulans* (200 mg/kg), *F. cretica* (200 mg/kg)	↓ Uric acid, reduced inflammation, improved gut microbiota	Upregulation of ABCG2 and SLC2A9, inhibition of TLR4/MyD88/NF-κB, IL-17 pathways	[45]
	MCF-7 cell line and rats (treated with 80 mg DMBA/kg)	Aqueous extract	100, 200 and 250 µg/mL; 60 and 120 mg/kg	Tumor regression, antioxidant activity, improved body weight	Cytotoxic activity and ROS scavenging	[16]
	MCF-7, HepG2, Caco-2	Alcoholic extract	6.9 ± 0.53 µg/mL	strong cytotoxicity, COX inhibition, apoptosis induction	COX-1, COX-2 inhibition, caspase-9 activation, topoisomerase inhibition	[27]
	In vitro	Ethanolic extract	0.5 µg/mL to 1000 µg/mL	Highest antioxidant activity, extracts scavenged DPPH	Scavenging of secondary metabolites, flavonoids, polyphenols (such as kaempferol-3-rhamnoside)	[39]
	In vitro/biochemical assays	Ethanolic extract	200 µL/mL	Reduction in oxidative stress and lipid peroxidation	Increased expression of SOD, CAT, GPx, and HO-1 → restoration of redox balance and cellular protection	[41]
Anti-inflammatory	Experimentally induced ulcerative colitis in mice	Ethanolic extract	100, 200 and 400 mg/kg	↓ colon inflammation, tissue injury, and disease severity	Inhibition of NF-κB signaling → suppression of TNF-α, IL-1β, IL-6, COX-2, iNOS via blocking IKK activation, preventing of NF-κB nuclear translocation	[47]
	Animal model	Ethanolic extract	100, 200 and 400 mg/kg	Acute inflammation, chronic inflammation, neutrophil activity	MOSO and OA depend on the activation of glucocorticoid receptors (GC)	[49]
	Phytochemical/mechanistic review evidence	Aqueous methanolic ethanolic	Not specified	Anti-inflammatory and antioxidant potential	Enhancement of antioxidant enzymes (SOD, CAT, GPx), reduction in ROS, and overall suppression of inflammation-related signaling pathways	[50]
	Sprague Dawley rats	Unfractionated extract; solvent NS; plant part NS.	50, 100 and 200 mg/kg	Antinociceptive, anti-inflammatory, anticoagulant activities	Reduction in inflammation via histamine pathways, modulation of pain and coagulation pathways	[51]
Anti-microbial	In vitro	Ethanolic extract	Not specified *	Successful synthesis of highly crystalline, spherical Ag NPs with an average size of 16 nm. Significant antibacterial activity against *P. vulgaris*, *E. coli*, and *K. pneumoniae*. Ag NPs outperformed commercial ciprofloxacin in these strains.	Induction of ROS (30% higher than control), leading to oxidative stress, DNA/RNA damage, and inhibition of protein synthesis	[85]
	In vitro	Aqueous *F. cretica* extract used as a reducing/stabilizing agent for nanoparticle synthesis; plant part NS	Not specified *	Green-synthesized ZnO NPs showed strong antibacterial and antioxidant activity	Phenolic groups, amino acids, and amide linkages in the plant extract act as reducing and stabilizing agents to convert zinc acetate into ZnO NPs.	[88]
	In vitro	Unfractionated extract; solvent NS; plant part NS, gradient HPLC fractions	Not specified *	Identified compounds (gallic acid, quinic acid, liquirtigenin, rosmarinic acid), active against GI pathogens	The antibacterial effect is attributed to secondary metabolites identified in the bioactive fractions, such as gallic acid, rosmarinic acid, and liquirtigenin.	[32]

Abbreviation used: Bax, Bcl-2-associated X protein; Bcl-2, B-cell lymphoma 2; COX-2, Cyclooxygenase-2; CYP3A4, Cytochrome P450 3A4; DMBA, 7, 12-dimethylbenz[a]anthracene; DPP-4 inhibition, Dipeptidyl peptidase-4; GLP-1, Glucagon-like peptide-1; IL-1, Interleukin-1; MOSO, *M. Oleifera* seed oil; NS, Not specified; PI3K/Akt, Phosphoinositide 3-kinase; ROS, Reactive oxygen species; TGR5 activation, Takeda G-protein-coupled receptor 5; TNF, Tumor necrosis factor; UGT2B7, UDP-glucuronosyltransferase 2; ZnO NPs, Zinc oxide nanoparticle. * In these studies, the authors tested the extracts in bioassays without defining precise concentrations; ↓ expression or concentration downregulated; ↑ expression or concentration upregulated.

## Data Availability

No new data were created or analyzed in this study. Data sharing is not applicable to this article.

## Abbreviations

The following abbreviations are used in this manuscript:

ABCG2	ATP-binding cassette subfamily G member 2
ABTS	2,2′-Azino-bis(3-ethylbenzothiazoline-6-sulfonic acid)
AGEs	Advanced glycation end products
AgNPs	Silver nanoparticles
AMPK	Adenosine monophosphate-activated protein kinase
ARE	Antioxidant response element
Bax	Bcl-2-associated X protein
Bcl-2	B-cell lymphoma 2
BH4	Tetrahydrobiopterin
CAT	Catalase
CCl4	Carbon tetrachloride
COX-1	Cyclooxygenase-1
COX-2	Cyclooxygenase-2
CYP2E1	Cytochrome P450 2E1
CYP3A4	Cytochrome P450 3A4
DMBA	7,12-Dimethylbenz[a]anthracene
DNMT1	DNA methyltransferase 1
DNA	Deoxyribonucleic acid
DPP-4	Dipeptidyl peptidase-4
DPPH	2,2-Diphenyl-1-picrylhydrazyl
eNOS	Endothelial nitric oxide synthase
ER-β	Estrogen receptor beta
ERK	Extracellular signal-regulated kinase
FADD	Fas-associated death domain
FOXO3a	Forkhead box O3a
FRAP	Ferric reducing antioxidant power
GCL	Glutamate-cysteine ligase
GLP-1	Glucagon-like peptide-1
GLUT4	Glucose transporter type 4
GPx	Glutathione peroxidase
GSH	Reduced glutathione
GSH/GSSG	Reduced glutathione/oxidized glutathione ratio
GSS	Glutathione synthetase
GSSG	Oxidized glutathione
HbA1c	Glycated hemoglobin
HCC	Hepatocellular carcinoma
HCEC	Human corneal epithelial cells
HepG2	Human hepatocellular carcinoma cell line
HO-1	Heme oxygenase-1
HPLC	High-performance liquid chromatography
IC50	Half-maximal inhibitory concentration
IKK	IκB kinase
IL-1β	Interleukin-1 beta
IL-6	Interleukin-6
IL-17	Interleukin-17
iNOS	Inducible nitric oxide synthase
IRS-1	Insulin receptor substrate-1
JNK	c-Jun N-terminal kinase
Keap1	Kelch-like ECH-associated protein 1
MAPK	Mitogen-activated protein kinase
MCF-7	Michigan Cancer Foundation-7 breast cancer cell line
MDA	Malondialdehyde
MDR	Multidrug-resistant
MMP	Matrix metalloproteinase
MPO	Myeloperoxidase
MTT	3-(4,5-Dimethylthiazol-2-yl)-2,5-diphenyltetrazolium bromide
MyD88	Myeloid differentiation primary response 88
NADPH	Nicotinamide adenine dinucleotide phosphate
NAFLD	Non-alcoholic fatty liver disease
NASH	Non-alcoholic steatohepatitis
NF-κB	Nuclear factor kappa-light-chain-enhancer of activated B cells
NO	Nitric oxide
NOX2	NADPH oxidase 2
NOX4	NADPH oxidase 4
NQO1	NAD(P)H quinone oxidoreductase 1
Nrf2	Nuclear factor erythroid 2-related factor 2
ONOO−	Peroxynitrite
PC3	Human prostate cancer cell line
PI3K/Akt	Phosphoinositide 3-kinase/protein kinase B pathway
PTEN	Phosphatase and tensin homolog
PTP1B	Protein tyrosine phosphatase 1B
RAGE	Receptor for advanced glycation end products
RNA	Ribonucleic acid
RNS	Reactive nitrogen species
ROS	Reactive oxygen species
SAR	Structure–activity relationship
SeNPs	Selenium nanoparticles
SMAD	Small mothers against decapentaplegic protein
SOD	Superoxide dismutase
STZ	Streptozotocin
TFC	Total flavonoid content
TGF-β	Transforming growth factor beta
TGR5	Takeda G-protein-coupled receptor 5
TLR4	Toll-like receptor 4
TNF-α	Tumor necrosis factor alpha
TPC	Total phenolic content
TRAIL	Tumor necrosis factor-related apoptosis-inducing ligand
Trx	Thioredoxin
UGT2B7	UDP-glucuronosyltransferase 2B7
VCAM-1	Vascular cell adhesion molecule-1
ZnO NPs	Zinc oxide nanoparticles
4-HNE	4-Hydroxynonenal
8-OHdG	8-Hydroxy-2′-deoxyguanosine
